# Muscle Strength Outcomes After ACL Reconstruction Before, During, and After COVID-19-Related Rehabilitation Disruptions

**DOI:** 10.3390/jcm14082751

**Published:** 2025-04-16

**Authors:** Martin Rudolf Zore, Nevenka Kregar Velikonja, Mohsen Hussein

**Affiliations:** 1 Artros Reha, Tehnološki park 22a, 1000 Ljubljana, Slovenia; mzore@artros.si; 2 Institute of Cellular Regenerative Medicine, Tehnološki park 22a, 1000 Ljubljana, Slovenia; 3 Department of Health Sciences, Alma Mater Europaea University, Slovenska 17, 2000 Maribor, Slovenia; 4 Faculty of Health Sciences, University of Novo Mesto, Na Loko 2, 8000 Novo Mesto, Slovenia; nevenka.kregar-velikonja@uni-nm.si; 5 Educell, Prevale 9, 1236 Trzin, Slovenia

**Keywords:** anterior cruciate ligament reconstruction, COVID-19, muscle recovery, strength testing

## Abstract

**Background:** Healthcare restrictions on non-urgent services during the COVID-19 pandemic led to limited access to rehabilitation and delayed treatment, potentially affecting early and mid-term recovery following anterior cruciate ligament reconstruction (ACLR). However, little is known about its specific consequences on muscle strength recovery in recreational and amateur athletes. **Objectives:** This study aimed to compare short-term clinical outcomes in ACLR patients before, during, and after the pandemic, spanning from 2020 to 2022. **Methods:** We conducted a retrospective review of 126 patients who underwent ACLR using a hamstring tendon autograft. Patients were grouped based on the timing of surgery and matched for gender. Clinical outcomes and muscle strength parameters were evaluated using an isokinetic dynamometer. **Results:** Male patients exhibited no significant differences in muscle strength across all time frames (control, 2020, 2021, and 2022). In contrast, female patients who underwent surgery in 2020 and 2021 showed higher extension asymmetry index deficits (Q-AI: 34.09 ± 14.59% and 36.47 ± 16.36%, respectively) and increased flexion deficits in 2021 (H-AI: 25.14 ± 11.41%). Significant differences were observed in quadriceps and hamstring peak torque values, both absolute and normalized. By 2022, the female group exhibited a reduced extension deficit (Q-AI: 18.64 ± 14.49%) comparable to pre-pandemic levels (Q-AI: 19.84 ± 12.37%), indicating a recovery of knee extensor strength. **Conclusion:** Our study showed gender-specific differences in muscle strength recovery after ACLR during the COVID-19 pandemic, with females showing poorer outcomes than males at 5 months post-operation. Larger studies with extended follow-up are needed to clarify the pandemic’s impact and gender-specific responses.

## 1. Introduction

Anterior cruciate ligament (ACL) injuries are common in active individuals, and surgical reconstruction (ACLR) aims to restore knee stability and prevent complications, such as instability, meniscal damage, and cartilage wear [1]. However, ACL re-injury rates remain high, with up to 22% for the operated knee and 24% for the contralateral knee within five years [2,3,4,5,6]. Effective postoperative rehabilitation is essential to address physical and psychological impairments, restore knee function, and enable a safe return to sports.

Persistent muscle weakness after ACLR is a major barrier to full recovery and increases re-injury risk [7,8,9]. Muscular weakness can be broadly defined as a reduction in the ability to produce maximal tension (peak torque, maximal strength). The degree of weakness is usually defined as the difference or asymmetry between the limbs and is most commonly calculated as the limb symmetry index (LSI)—the difference between the peak torque of the ACLR limb and the uninvolved limb (i.e., LSI (%) = (injured limb/non-injured limb) × 100) or an asymmetry index (AI) (e.g., AI (%) = (non-injured limb − injured limb)/(non-injured limb) × 100). An AI of knee extensor muscle strength between ACLR and the uninjured knee greater than 20% (6 months after ACLR) predicts significant differences in the strengths of both legs even two years after ACLR [10,11]. Severe asymmetry in quadriceps femoris muscle strength and its weakness after primary anterior cruciate ligament reconstruction (ACLR) prevents return to increased sports activity, is associated with poorer patient satisfaction and function of the operated knee, and results in altered lower limb biomechanics during movement. Therefore, muscle strength of the relevant dynamic knee joint stabilizers is an important risk factor for secondary ACL injury [7,12,13,14].

The COVID-19 pandemic, declared by the World Health Organization in March 2020, led to widespread public health measures, including the closure of fitness centers and sports fields, as well as the suspension of non-urgent healthcare services, such as elective surgeries. These restrictions disrupted access to in-person postoperative rehabilitation and gym-based strength training. Healthcare delivery was heavily impacted by COVID-19 restrictions, with in-person services limited to essential needs under strict safety protocols during the initial closures. Telehealth became the primary mode for rehabilitation, and routine in-person care only gradually resumed under controlled conditions starting in June 2020. However, new waves of COVID-19 cases continued to disrupt services, requiring ongoing adaptations and safety measures. Full rehabilitation services and gym access were not widely available until after mass vaccinations in mid-2021, which allowed for fewer restrictions.

Throughout this period, ACLR rehabilitation faced major challenges. The first wave caused the most significant disruptions, but subsequent waves also demanded flexibility from healthcare providers and patients. A persistent obstacle was the closure of gyms and fitness centers, which limited access to equipment essential for achieving adequate loading in middle and later stages of ACLR rehabilitation, potentially compromising recovery outcomes.

The full extent of the consequences of the pandemic on the short- and long-term patients’ outcomes are not yet known. Kopka et al. (2020) studied patient-reported outcomes and found that 41% of the patients whose surgery was postponed had increased symptoms related to their injuries [15]. More than 71% of early postoperative patients reported that closures negatively impacted their recovery, and more than 62% of patients whose surgery was postponed described a negative impact on their ability to return to work and sports [15].

There are some reports of good short-term subjective outcomes in patients who underwent ACLR just before or during the first months of the pandemic. Patients were able to maintain personal contact with their health professionals, coped well, and had a positive outlook. However, there is a lack of short- and long-term studies examining objective impairments, such as muscle strength, which could lead to significant long-term complications after ACLR surgery (increased risk profile for second injury) during and after the pandemic.

A retrospective cohort study by Weaver et al. (2022) was the only study of which we are aware that compared short-term (3 months) outcomes of quadriceps and hamstring muscle strength and function in adolescents after ACLR for those who had surgery before COVID-19 versus during the pandemic [16]. The study reported that the only significant difference between groups was found in the normalized quadriceps peak torque at the uninvolved limb, with the control group (2.03 ± 0.47 Nm/kg) having lower peak torque compared to the COVID-19 group (2.49 ± 0.61 Nm/kg) (*p* = 0.002, effect size (d) = 0.84). No differences were found between the groups for any of the other strength outcomes [16].

Given the central role of progressive resistance training in restoring muscle strength, preventing re-injury, supporting a safe return to sports, and maintaining long-term knee health, it is crucial to understand how pandemic-related constraints may have influenced recovery. Beyond the suspension of non-urgent healthcare services, the COVID-19 pandemic introduced broader societal disruptions—including mandatory isolation, movement restrictions, reduced social interaction, and physical distancing—which collectively reduced access to supervised rehabilitation and equipment. These factors particularly affected the structured, progressive loading required in the early and mid-stages of ACLR rehabilitation. Patients were often forced to rely on unsupervised home-based programs or telehealth check-ins, which may have compromised the adherence to, precision of, and progression of exercise. These unique conditions may have affected subgroups differently, who may face greater physical and psychological vulnerabilities post-ACLR. This study aimed to evaluate the consequences of pandemic-related restrictions at different phases (2020 and 2021), including the post-pandemic period (2022), on short-term rehabilitation outcomes after ACL reconstruction (ACLR). Specifically, the study aimed to investigate (1) how pandemic-related restrictions affected muscle strength recovery in men and women and (2) whether short-term clinical outcomes differed in those who underwent ACLR surgery and rehabilitation before COVID-19 versus during the COVID-19 pandemic. The authors hypothesized that limited access to rehabilitation facilities and the general closure of sports and gymnasium facilities during the pandemic would have a significant indirect negative impact on patient recovery, both in males and females, and that the COVID-19 groups (2020 and 2021) would have worse muscle strength outcomes, including a lower quadriceps limb asymmetry index (Q-AI) and higher H/Q ratios, than the pre-pandemic group and the post-COVID-19 group (2022).

## 2. Materials and Methods

### 2.1. Study Design and Ethics

A retrospective review of the records of patients after ACL injury and ACLR in pre-pandemic, pandemic, and post-pandemic periods was performed. All patients were treated in the same institution and by the same surgeon. During the observation period, a total of 126 patients who underwent ACLR using semitendinosus and gracilis tendon autografts were included; this technique is the predominantly used method for optimizing knee stability and minimizing complications in the institution. The preoperative evaluation of patients included demographic and anthropometric data such as age, gender, height, weight, BMI, and the preoperative Tegner activity score. Patients were divided into four groups according to the period of the treatment. Data were analyzed separately for each gender. This study was approved by the Institutional Review Board (R-42022); all included patients participated in agreement with the Declaration of Helsinki and gave voluntary informed consent.

### 2.2. Study Sample

In the 2020 group, 26 patients underwent ACLR surgery, of whom 22 met the inclusion criteria. In the 2021 group, 31 patients underwent surgery, of whom 27 met the inclusion criteria. In the 2022 group, 43 patients underwent surgery, of whom 27 met the inclusion criteria. The control group of 50 patients was randomized from the cohort of 350 patients that underwent surgery between April 2016 and November 2019 (pre-pandemic group). The follow-up of the control group did not overlap with that of the COVID-19 group.

### 2.3. Inclusion and Exclusion Criteria

The inclusion criteria for the patients were as follows: (1) they underwent primary ACLR reconstruction with semitendinosus and gracilis autografts, (2) they completed a standardized isokinetic strength test at 60 deg/s and a clinical orthopedic examination, and (3) all injuries occurred during pivoting sports, including tennis, badminton, soccer, and basketball, with mechanisms involving non-contact or indirect contact events. The postoperative period after ACLR testing was not an inclusion criterion because COVID-19 periods could vary considerably due to COVID-19 social isolation measures. The correlation coefficient was used to test whether the timing of examination after ACLR had an influence on outcome variables (limb asymmetry index, H/Q ratio, and normalized peak torque to body weight). The exclusion criteria were (1) multiligamentous injury, (2) severe arthritic changes of the knee evaluated as a grade III-IV Kellgren–Lawrence (KL) or grade III-IV ICRS cartilage injury (International Cartilage Regeneration and Joint Society), (3) total or subtotal meniscectomy prior to ACLR on either side, and (4) contralateral ACL deficient knee.

### 2.4. Patients and Treatment Procedures

Patients attended an educational physiotherapy session before surgery and were instructed to perform a home rehabilitation program while awaiting ACLR to address any existing impairments, including effusion, range of motion, pain, gait issues, and muscle weakness.

The rehabilitation program was structured into four phases according to Brukner and Khan (2021) [17], and all patients included in this study were instructed to follow the same protocol, regardless of the situation with the pandemic. Phases 1 and 2 included two physiotherapy sessions per week during the first five weeks after ACLR. Phase 3 began at week 10 after ACLR and included two weeks of intensive postoperative rehabilitation with daily sessions at a rehabilitation center following the standard rehabilitation protocol, which included exercise therapy in a swimming pool and a strengthening program in a gym. The primary objective during this phase was to educate and guide patients on the correct execution, dosage, and progression of exercises.

However, the implementation of the rehabilitation program was significantly affected by the COVID-19 restrictions and closures that applied from March 2020 to early June 2020, which was the most stringent period. During this period, all non-essential health services, including physiotherapy for postoperative rehabilitation and fitness centers, had to be closed. Consequently, patients who underwent surgery in early 2020 faced a three-month delay in the phase 2 and 3 rehabilitation process. During this time, they received online consultations with physiotherapists once every three weeks and were provided with a home rehabilitation program.

The second and third closures then occurred from October 2020 to the end of February 2021 and again in April 2021 for three weeks. Despite these closures, personal physiotherapy for postoperative care was allowed to continue during phases 1 and 2 of the rehabilitation while maintaining strict safety precautions. However, all fitness and sport centers remained closed, which affected phases 3 and 4 of rehabilitation. Consequently, patients did not have access to the gym and received two versions of written instructions for the late-phase rehabilitation program, to be performed either at home or at an alternative gym until muscle strength testing with an isokinetic dynamometer was monitored.

Patients were grouped based on annual time frames, taking into account the delay in the start of rehabilitation and the adapted home rehabilitation program after ACLR in the years 2020 and 2021. The patient group in 2022 was already the post-pandemic group with no delay or restrictions in rehabilitation, as was the pre-pandemic control group, which had the same rehabilitation protocol as explained in the above section but without the limitations for the performance of the rehabilitation process.

### 2.5. Outcome Measures

Time intervals between surgery and postoperative testing were documented and calculated. Isokinetic, concentric–concentric knee extension, and flexion strength (peak torque) were measured bilaterally between 0° and 90° using a Humac Norm isokinetic dynamometer (Humac Norm Testing & Rehabilitation System; CSM Inc., Stoughton, MA, USA) at a speed of 60°/s, as used in other similar studies [18,19,20]. The asymmetry index was calculated (e.g., AI (%) = (non-injured limb − injured limb)/(non-injured limb) × 100). The testing procedure was identical between all groups. Knee extension and flexion strength testing using the Humac Norm dynamometer was reliable when measuring the isokinetic muscle strength (Intraclass Correlation Coefficient = 0.89).

To reduce potential sources of bias, patient groups were matched by gender and surgery timing. A retrospective review of records was conducted, with two independent clinicians collecting data for the pandemic years (2020 and 2021) to ensure accuracy. Data for other years were collected by the first author of the paper. This comprehensive approach aimed to enhance the robustness of our findings by considering potential biases during data collection.

### 2.6. Statistical Analysis

The knee peak torques, H/Q ratios, and normalized peak torques to body weight (Nm/kg) of knee muscles were analyzed in order to find statistically significant differences among patients, treated at different time frames. All results were reported as the mean and standard deviation (SD). Firstly, the measurement data were assessed for normality of distribution using the Shapiro–Wilk test. This test was performed separately for the male and female datasets, considering gender-specific variations in the distribution of the variables. The results indicated that both groups exhibited a normal distribution of measured variables, allowing further parametric statistical analysis. Kruskal–Wallis one-way analysis of variance (ANOVA) was used for testing whether there was a statistically significant difference in the assessment of demographic characteristics and measured parameters among time frame groups (control group, 2020 group, 2021 group, and 2022 group). The LSD post hoc test allowed for a detailed interpretation of significant differences between the mean values of the variables. The level of statistical significance was set at *p* < 0.05. Statistical analysis was performed using SPSS (version 25, IBM Corporation Armonk, New York, NY, USA).

## 3. Results

### 3.1. Baseline Patient Characteristics

Demographic characteristics, as well as physical and activity measurements taken during preoperative patient evaluations, are presented in Table 1.

The study included a total of 126 patients who met the inclusion criteria, comprising 70 males (56%) and 56 females (44%). As mentioned in the Section 2 of the article, the patients were grouped based on yearly timeframes. No significant differences were observed among the compared groups in terms of demographic characteristics and other relevant variables, including age, gender, Tegner activity, body mass, body height, body mass index, and time from surgery to testing. The mean age of female patients (36.45 ± 12.56) was higher than that of male patients (33.81 ± 11.14), although the difference was not statistically significant (*p* = 0.222). This indicates that the groups were comparable in terms of these specific factors, minimizing the potential confounding effects that could arise from differences in these variables. The testing period for all groups spanned between 20.74 weeks and 28.61 weeks after surgery, as detailed in Table 1.

### 3.2. Muscle Strength Testing Results

There were no significant differences among time frame groups (control, 2020, 2021, and 2022 groups) in any measured parameter in male patients (Table 2). However, in female patients, the measurements revealed a significantly higher extension deficit (Q-AI, 36.47%) and flexion deficit (H-AI, 25.14%) in the group that was operated on in 2021 (Table 3). ANOVA showed significant differences in quadriceps and hamstring peak torque values and normalized peak torque values. In female patients, a reduction in extension deficit (Q-AI, 18.64) was observed in the 2022 group that was lower than the pre-pandemic normative value in the control group (Q-AI, 19.84%), indicating an improvement in clinical outcomes of knee extensor muscle strength (Q-AI), compared to pre-pandemic levels in 2022.

Additionally, Table 4 presents the hamstring-to-quadriceps (H/Q) ratios for both the ACLR and uninvolved limbs across all time frames. No statistically significant differences were observed between groups for either gender, indicating that the H/Q ratio remained relatively stable despite rehabilitation disruptions.

## 4. Discussion

The aim of this study was to investigate potential differences in muscle strength outcomes among male and female patients following ACL reconstruction (ACLR) across different timeframes affected by the COVID-19 pandemic. Specifically, outcomes were compared between the pre-pandemic period, the pandemic years (2020 and 2021), and the post-pandemic year (2022). Our findings indicate that female patients who underwent ACLR during the pandemic experienced significantly poorer muscle strength recovery compared to those treated before or after the pandemic. In contrast, no significant differences were observed among male patients across any timeframe. These results suggest a gender-specific vulnerability to the rehabilitation constraints imposed by pandemic-related restrictions.

This study adds to the existing body of literature by focusing on objective measures of muscle strength, whereas most prior studies have relied on subjective or patient-reported outcomes [15,21,22]. To our knowledge, this was the first study to assess quadriceps and hamstring limb symmetry (Q-AI and H-AI) outcomes across pre-pandemic, pandemic, and post-pandemic timeframes using sex-disaggregated data. Moreover, our study is unique in utilizing concentric isokinetic muscle strength testing of both knee flexors and extensors. In contrast, Weaver et al. (2022) used isometric testing only and did not report hamstring-to-quadriceps (H/Q) ratios, which limits the comparability of the findings [16]. Their results, which showed no differences in strength outcomes during the pandemic, were also not broken down by gender, possibly masking gender-specific effects.

Notable differences were observed between male and female groups. Male patients did not appear to be affected by the pandemic, showing no significant differences in the measured parameters across any of the time frame groups (control, 2020, 2021, and 2022) (Table 2). In contrast, female patients had worse Q-AI and H-AI outcomes during the pandemic years (2020 and 2021) (Table 3).

Other literature has highlighted that healthy males have a 1.5- to 2-times greater muscle strength across age groups [23]. Moreover, research shows that females are 2 to 8 times more likely to suffer from ACL injuries, compared to males [24]. In addition, the risk of experiencing contralateral ACL injury is 33% higher in women than in men [25]. This increased risk is thought to be influenced by a combination of various factors, including anatomic, biomechanical, neuromuscular, and environmental factors.

Several reports suggest that women may have decreased quadriceps strength and greater extension range of motion (ROM) deficits at 3 and 6 months after ACLR. Factors such as female gender, higher BMI, and loss of extension ROM have been identified as independent predictors of worse quadriceps strength 6 months after surgery [26]. Our findings are consistent with the study published by Van Melick et al. (2022), which showed that female patients have lower Q-AI and lower normalized peak torque relative to body mass (Nm/kg) for quadriceps and hamstring muscle groups, compared with male patients [20].

Furthermore, gender differences in return to pre-injury sport levels, perceived instability, psychological distress, and sociocultural factors (e.g., social roles, psychological responses, and rehabilitation experiences) following ACL injury and recovery have been reported. A recent systematic review and meta-analysis by Bruder et al. (2023) identified notable gender differences in self-reported activity and knee-related outcomes after ACL injury and reconstruction [27]. The findings suggest that female athletes tend to experience higher psychological distress and report lower activity levels and worse knee-related outcomes compared to their male counterparts. Although the data are limited and uncertain, available evidence indicates that female athletes are approximately 25% less likely to return to sports within five years of ACL injury or reconstruction compared to males [27].

During the pandemic, these vulnerabilities may have been further amplified. The impact of the COVID-19 pandemic and its containment measures on mental health in the general population, particularly in relation to gender differences, is still largely unknown. The study of Kregar Velikonja et al. (2020) revealed significantly higher psychological burden and anxiety scores in women than in men in the beginning of the pandemic [28]. Salanti et al. (2022) found that after an initial increase in average symptoms of depression and anxiety, along with a correlation between stricter measures and higher reported cases, there were substantial variations in mental health symptoms observed in studies conducted beyond the first two months of the pandemic [29]. These findings highlight the diverse responses of different populations to the psychological stress induced by the pandemic and its containment measures. The current literature lacks sufficient research to determine whether females who underwent orthopedic procedure or ACLR during the pandemic period were more prone to psychological distress that could influence behaviors that hindered their compliance with long-distance guided rehabilitation programs.

Our study found that the pandemic-related restrictions had a significant indirect negative impact on the recovery of female patients in the early stages of rehabilitation but not on male patients. The results showed no significant differences in muscle strength outcomes between patients who underwent ACLR before and during the COVID-19 pandemic in male patients. This suggests that male patients were able to achieve similar muscle strength recovery outcomes despite challenges during the pandemic.

These findings have practical implications. Clinicians should be aware of the potential for disproportionate recovery setbacks in female patients during times of limited rehab access. Future rehabilitation strategies should consider gender-specific needs and may include closer monitoring, proactive psychological support, and adapted remote rehab programs for vulnerable populations.

It is important to consider the limitations of this study. While the study provides valuable insights, considerations of population heterogeneity and surgical techniques are necessary for broader generalizability. The sample size was relatively small, particularly among female subgroups, which limited statistical power and may have reduced our ability to detect medium-sized effects. This was partly due to a reduced number of ACLR procedures during the COVID-19 pandemic, when elective surgeries were restricted. Although we considered a post hoc power analysis, post hoc power is widely viewed as uninformative and potentially misleading [30]. Hence, our statistical interpretation relies primarily on *p*-values from ANOVA (Kruskal–Wallis) and descriptive metrics (mean, SD), supplemented by LSD post hoc tests. Consequently, these findings should be regarded as preliminary, and larger, prospectively powered studies are required to confirm them. In addition, the retrospective design prevented precise control over potential confounders (e.g., adherence, mental health), which may have influenced the observed outcomes.

All patients adhered to a standardized rehabilitation protocol; however, protocol implementation was influenced by the COVID-19 pandemic, resulting in variations in access to gyms and rehab facilities. These variations were attributed to factors such as gym closures and limited access to fitness equipment, individual adherence to the rehabilitation program, level of self-efficacy, motivation, and other relevant factors. Although no data were collected on urban or rural residence, which is a limitation of this study, we verified that all patients lived within 40 km of the rehabilitation center, reducing the likelihood of significant geographic barriers. Future studies should include place of residence to better assess its influence on rehabilitation outcomes. Additionally, the study assessed only short-term outcomes, and the long-term effects of the pandemic on muscle strength recovery are not yet known. Further studies with larger samples and longer follow-up periods are needed to fully understand the impact of the limited access to rehabilitation centers due to the pandemic or other reasons on ALCR outcomes and differences in muscle strength outcomes between men and women.

## 5. Conclusions

The study found gender differences in muscle strength outcomes after ACLR in different time periods influenced by the COVID-19 pandemic. Female patients had worse outcomes in the pandemic years (2020 and 2021), while the pandemic did not affect the recovery of male patients. In the post-pandemic period (2022), this influence on female patients was not observed anymore.

Overall, this study contributes to the existing literature by providing objective measures of muscle strength outcomes after ACLR during the pandemic. It highlights the need for a tailored rehabilitation approach and underscores the importance of considering gender differences in ACLR outcomes. By addressing these issues, healthcare professionals can improve treatment strategies and optimize muscle strength recovery in both male and female patients undergoing ACLR.

## Figures and Tables

**Table 1:** Patient demographic data.

	All		Control Group		2020		2021		2022		
Factor	Mean (SD)		Mean (SD)		Mean (SD)		Mean (SD)		Mean (SD)		*p*-Value (ANOVA)
Age—Male (yrs)	33.81	(11.14)	34.20	(11.10)	34.47	(11.74)	33.91	(10.56)	32.59	(12.23)	0.964
Age—Female (yrs)	36.45	(12.56)	36.54	(11.42)	36.10	(12.51)	34.36	(12.65)	38.90	(16.33)	0.881
Body Mass—Male (kg)	85.00	(16.41)	86.85	(18.19)	80.67	(10.76)	80.01	(12.79)	89.94	(18.92)	0.244
Body Mass—Female (kg)	61.89	(10.58)	61.83	(10.15)	61.60	(9.54)	63.91	(13.77)	60.10	(9.89)	0.881
Body Height—Male (cm)	1.80	(0.07)	1.81	(0.08)	1.80	(0.06)	1.79	(0.06)	1.81	(0.08)	0.846
Body Height—Female (cm)	1.66	(0.06)	1.67	(0.06)	1.64	(0.05)	1.67	(0.05)	1.66	(0.06)	0.629
Body Mass Index—Male (kg/m^2^)	26.07	(4.56)	26.51	(5.35)	24.84	(2.33)	24.97	(4.09)	27.32	(4.75)	0.350
Body Mass Index—Female (kg/m^2^)	22.31	(3.24)	22.11	(2.57)	22.74	(2.92)	22.82	(4.86)	21.81	(3.25)	0.865
Tegner Activity Score—Male	7.17	(1.13)	7.00	(0.89)	7.33	(1.50)	7.06	(0.85)	7.41	(1.42)	0.635
Tegner Activity Score—Female	6.45	(1.07)	6.58	(1.10)	6.20	(1.23)	6.09	(0.70)	6.80	(1.14)	0.364
Time from surgery to testing—Male (weeks)	22.54	(4.73)	22.86	(4.49)	24.05	(7.68)	22.68	(3.44)	20.84	(3.11)	0.357
Time from surgery to testing—Female (weeks)	22.80	(7.00)	21.68	(2.77)	20.74	(3.49)	21.82	(3.45)	28.61	(14.22)	0.165
Gender, Male/Female, n	70/56		26/24		12/10		16/11		16/11		

Indicates significant difference (*p* ≤ 0.05).

**Table 2:** Mean Values of Strength and symmetry for knee extension and flexion in different time frames. All data are presented as the mean (SD)—male.

		Extension					Flexion				
		ACLR	Nm/kg	Uninvolved	Nm/kg	Q-AI	ACLR	Nm/kg	Uninvolved	Nm/kg	H-AI
Group	N										
Control	26	170.46	2.00	218.62	2.6	23.20%	129.77	1.5	146.77	1.7	11.80%
		(56.60)	(0.7)	(47.56)	(0.5)	(18.4)	(33.80)	(0.40)	(32.08)	(0.40)	(10.90)
2020	12	174.67	2.2	229.00	2.9	22.50%	136.25	1.7	144.67	1.8	5.60%
		(38.51)	(0.50)	(32.56)	(0.40)	(19.10)	(22.91)	(0.30)	(22.72)	(0.30)	(10.00)
2021	16	165.94	2.1	223.56	2.8	26.10%	121.63	1.55	140.25	1.78	13.99%
		(37.57)	(0.50)	(34.51)	(0.40)	(9.68)	(27.17)	(0.38)	(18.02)	(0.31)	(11.53)
2022	16	174.18	2.00	221.82	2.55	22.98%	121.82	1.41	147.59	1.69	16.91%
		(64.55)	(0.70)	(46.56)	(0.65)	(16.16)	(31.60)	(0.44)	(33.05)	(0.39)	(16.12)
*p* value		0.965	0.809	0.189	0.544	0.930	0.509	0.257	0.874	0.776	0.114

*p*-value determined using ANOVA. ACLR = quadriceps (extension) or hamstrings (flexion) peak torque of the operated knee, uninvolved = quadriceps (extension) or hamstrings (flexion) peak torque of the uninvolved knee, Nm/kg = normalized peak torque to body mass, Q-AI = quadriceps limb asymmetry index, H-AI = hamstring limb asymmetry index.

**Table 3:** Mean values of strength and symmetry for knee extension and flexion in different time frames. All data are presented the mean (SD)—female.

		Extension					Flexion				
		ACLR	Nm/kg	Uninvolved	Nm/kg	Q-AI	ACLR	Nm/kg	Uninvolved	Nm/kg	H-AI
Group	N										
Control	24	109.83	1.79	136.92	2.23	19.84	87.33	1.43	94.83	1.55	7.19
		(27.60)	(0.42)	(27.83)	(0.39)	(12.37)	(13.86)	(0.25)	(16.73)	(0.28)	(9.97)
2020	10	88.30	1.48	137.10	2.26	34.09	78.70	1.30	87.20	1.44	9.65
		(20.20)	(0.44)	(27.62)	(0.47)	(14.59)	(12.68)	(0.27)	(12.30)	(0.28)	(10.52)
2021	11	84.18	1.38	133.64	2.14	36.47	67.00	1.09	89.82	1.45	25.14
		(26.50)	(0.52)	(26.58)	(0.48)	(16.36)	(12.73)	(0.31)	(12.77)	(0.28)	(11.41)
2022	11	106.90	1.82	133.90	2.28	18.64	81.00	1.38	91.60	1.56	9.29
		(28.09)	(0.57)	(35.13)	(0.71)	(14.49)	(15.46)	(0.34)	(25.26)	(0.47)	(12.58)
*p* value		0.027 *†	0.049 †#	0.984	0.918	0.002 *†#§	0.002 †§	0.014 †#	0.662	0.692	<0.001 †‡§

* *p*-value determined using ANOVA. Statistically significant difference between control and 2020, † statistically significant difference between control and 2021, ‡ statistically significant difference between 2020 and 2021, # statistically significant difference between 2020 and 2022, § statistically significant difference between 2021 and 2022. ACLR = quadriceps (extension) or hamstrings (flexion) peak torque of the operated knee, uninvolved = quadriceps (extension) or hamstrings (flexion) peak torque of the uninvolved knee, Nm/kg = normalized peak torque to body mass, Q-AI = quadriceps limb asymmetry index, H-AI = hamstring limb asymmetry index.

**Table 4:** Mean values (SD) of H/Q ratios in time frame groups for males and females.

		Male		Female	
Group		ACLR H/Q Ratio	Uninvolved H/Q Ratio	ACLR H/Q Ratio	Uninvolved H/Q Ratio
Control		0.83	0.68	0.82	0.70
	SD	(0.27)	(0.09)	(0.15)	(0.09)
2020		0.82	0.63	0.93	0.65
	SD	(0.22)	(0.04)	(0.28)	(0.08)
2021		0.74	0.64	0.88	0.69
	SD	(0.10)	(0.09)	(0.39)	(0.10)
2022		0.74	0.67	0.78	0.70
	SD	(0.21)	(0.08)	(0.13)	(0.17)
*p* value		0.507	0.257	0.464	0.585

ACLR H/Q ratio = ratio between hamstrings (flexion) and quadriceps (extension) peak torque of the operated knee, uninvolved H/Q ratio = ratio between hamstrings (flexion) and quadriceps (extension) peak torques of the uninvolved knee.

## Data Availability

The data presented in this study are available upon request from the corresponding author.
